# Levocetirizine Pretreatment Mitigates Lipopolysaccharide-Induced Lung Inflammation in Rats

**DOI:** 10.1155/2018/7019759

**Published:** 2018-08-13

**Authors:** Alaa N. A. Fahmi, George S. G. Shehatou, Hatem A. Salem

**Affiliations:** Department of Pharmacology and Toxicology, Faculty of Pharmacy, Mansoura University, Mansoura, Egypt

## Abstract

This research was conducted to investigate possible protective influences of levocetirizine, a nonsedating H_1_ antihistamine, against lipopolysaccharide (LPS)-induced lung injury in rats. Male Sprague Dawley rats received either levocetirizine (1 mg/kg/day, orally) or the vehicle of the drug (2 ml/kg/day, orally) for 1 week before a single IP injection of LPS (7.5 mg/kg). A group of normal rats served as control. The experiments were terminated 18 h after the LPS challenge. Serum C-reactive protein levels were determined. Moreover, total cell count, lactate dehydrogenase (LDH) activity, protein levels, and total NOx were evaluated in bronchoalveolar lavage fluid (BALF). Pulmonary edema was evaluated as the wet/dry lung weight ratio. Lung tissue homogenate was assessed for antioxidant/pro-oxidant status. BALF and lung tissue levels of tumor necrosis factor-*α* (TNF-*α*) were assessed. Lungs were examined for histological alterations. LPS-mediated lung injury was manifested by pulmonary edema, leukocyte infiltration, oxidative stress, and inflammation. Levocetirizine attenuated lung edema and mitigated the increases in BALF protein levels, LDH activity, and lung leukocyte recruitment in LPS-challenged rats. Additionally, TNF-*α* protein levels in BALF and lung tissue were diminished by levocetirizine administration. Levocetirizine also exhibited a potent antioxidant activity as indicated by a decrease in lung tissue levels of malondialdehyde and an enhancement of superoxide dismutase activity. Histological examination of lung tissues confirmed the beneficial effect of levocetirizine against LPS-induced histopathological alterations. In conclusion, levocetirizine may offer protection against lung tissue damage and inflammation in LPS-challenged rats.

## 1. Introduction

Acute lung injury (ALI) is a critical pathological event that causes acute pulmonary failure and death. On the clinical ground, the term ALI has been widely replaced with the acute respiratory distress syndrome (ARDS), which is currently defined based on several diagnostic criteria [1]. The mortality of patients suffering from ARDS remains high, around 30-50%, despite substantial advances in intensive care [2]. The ALI/ARDS may arise due to a diverse set of inciting insults such as major trauma, burns, pneumonia, aspiration, and sepsis [1]. Of these factors, sepsis related to bacterial infection garners special interest since it remains the most common etiology of postsurgery and posttrauma deaths [3].

Lipopolysaccharide (LPS) is a component of the cell wall of gram negative bacteria. Its release is the main etiological factor of bacterial endotoxemia and sepsis, which are associated with sequential dysfunction of multiple organs, including lung, heart, liver, and kidney [4–6]. LPS is robustly used to elicit experimental ALI, which exhibits major features of lung tissue injury in human ARDS, including leukocyte infiltration, lung edema, abnormal gas exchange, and mortality [7, 8]. Potential contributing mechanisms include disruption of alveolar-capillary membrane integrity and excessive neutrophil infiltration into alveolar spaces [9]. Lung inflammation also triggers the generation of various inflammation-associated cytokines and reactive oxygen species (ROS) [7, 10]. Moreover, apoptosis takes place in several cell types that are located in the inflammatory lung milieu [7].

Histamine H_1_-receptor antagonists represent a well-established therapeutic strategy for treatment of allergic and inflammatory diseases [11, 12]. The inflammation-inhibiting effects of H_1_-receptor blockers have been linked to both H_1_-receptor-dependent [13–15] and independent [15–17] mechanisms. This study was aimed to elucidate the influences of levocetirizine, a nonsedating H_1_ antihistamine, on LPS-induced lung tissue damage and inflammation in rats.

## 2. Materials and Methods

### 2.1. Animals

Male Sprague Dawley rats (190-230 g) were supplied with standard diet and water ad libitum. The experimental protocols in this study conformed to the institutional and international guidelines for the ethical use of laboratory animals in research.

### 2.2. Materials

LPS was obtained from Sigma Chemical Co. (E. coli serotype O111:B4, St. Louis, MO, USA). It was dissolved in sterile saline on the day of experiment. Levocetirizine (Levcet tablets, Marcryl co., Cairo, Egypt) was administered as a suspension in 0.5% carboxymethyl cellulose (CMC).

### 2.3. Experimental Protocol

Rats were assigned into 2 groups (n = 6 each) at random, as follows: LPS received 0.5% CMC (2 ml/kg/day for a week, orally) and Levocetirizine + LPS administered with levocetirizine (1 mg/kg/day for a week, orally). On day 8, both groups received LPS (7.5 mg/kg, IP) to induce ALI, as previously described [18, 19]. Control group (n = 6) received 0.5% CMC (2 ml/kg/day for a week, orally) and was injected with sterile saline (0.9%, IP) on day 8.

The selected dose of levocetirizine was previously reported in rat studies [16, 20]. At this dose, levocetirizine only elicits peripheral H_1_ blockade due to poor penetration into the CNS [21].

18 h after LPS challenge, blood was collected and serum was separated for measurement of C-reactive protein (CRP). The chest was surgically opened at the midline and the bronchoalveolar lavage fluid (BALF) was collected as previously described [18]. Moreover, lungs were removed, washed, and used for assessment of lung water content and histopathological examination.

In another series of experiments, the left lungs of treated rats were removed, homogenized (1:10 w/v) in 50 mM phosphate buffer (pH 7.4), and centrifuged (5000 rpm, 4°C, 20 min). Supernatants were then collected for assessment of oxidative status and tumor necrosis factor-*α* (TNF-*α*).

### 2.4. BALF Biochemical Parameters

Commercial assay kits for determination of total protein concentration (Biodiagnostic, Giza, Egypt), lactate dehydrogenase (LDH) activity (Biosystems, Barcelona, Spain), and total nitrite and nitrate (NOx) levels (R and D Systems, Minneapolis, USA) were used.

### 2.5. Lung Wet/Dry (W/D) Weight Ratio

The lower right lung lobe was weighed after isolation (wet weight) and after drying at 80°C for 24 h (dry weight). The W/D ratio was determined.

### 2.6. Lung Oxidative Stress

Levels of reduced glutathione (GSH), malondialdehyde (MDA), and superoxide dismutase (SOD) activity in lung homogenates were assayed using commercial kits (Biodiagnostic, Giza, Egypt), following manufacturer's instructions and based on previously reported methods [22–24].

### 2.7. TNF-*α* Levels

TNF-*α* concentrations in lung homogenates and BALF were determined using an ELISA kit (eBioscience, San Diego, CA), following manufacturer's instructions.

### 2.8. Histopathological Assessments

The upper right lung lobe was fixed in buffered formalin and stained with hematoxylin and eosin (H&E). Histological changes indicative of ALI were assessed, including alveolar congestion, hemorrhage, neutrophil infiltration, alveolar wall thickness, and interstitial edema. Tissues were graded on a scale of 0-3 for each characteristic to obtain a total lung injury score, as previously described [18]. Histological evaluation was carried out by a pathologist who was unaware of group assignment.

### 2.9. Statistical Analysis

Results are shown as mean ± SEM. Statistical comparisons were made using one-way analysis of variance (ANOVA) followed by Tukey–Kramer post hoc test. Kruskal-Wallis followed by Dunn's test was used to compare histological lung scores in the study groups. The level of significance was set at P < 0.05. Graphpad Prism software (V6.03, CA, USA) was used for statistical data analysis.

## 3. Results

### 3.1. Serum CRP Level (Figure 1)

Serum CRP levels were significantly higher in LPS-treated rats than control group (P < 0.0001), indicating an acute inflammation. Pretreatment of rats with levocetirizine restored normal serum level of CRP (P < 0.0001 compared to LPS group).

### 3.2. Lung W/D Weight Ratio (Figure 2)

The lung W/D weight ratio was significantly elevated in LPS group (P < 0.001) compared with control rats. Levocetirizine pretreatment attenuated LPS-induced elevation in the lung W/D ratio (P < 0.05 relative to LPS-challenged rats).

### 3.3. BALF Protein Content and Total Cell Count

**Figure 3:**
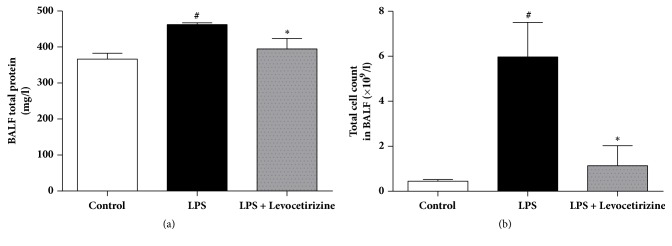
Effects of levocetirizine on protein content (a) and total cell count (b) in bronchoalveolar lavage fluid (BALF) of LPS-challenged rats. Results are shown as means ± SEM of 6 rats in each group. *∗* and # are significantly different (P < 0.05) versus LPS-treated and control groups, respectively.

BALF protein content (Figure 3(a)) and total cell count (Figure 3(b)) were significantly increased in rats after LPS administration, effects that were markedly attenuated by prior treatment with levocetirizine.

### 3.4. BALF Levels of LDH Activity and NOx

**Figure 4:**
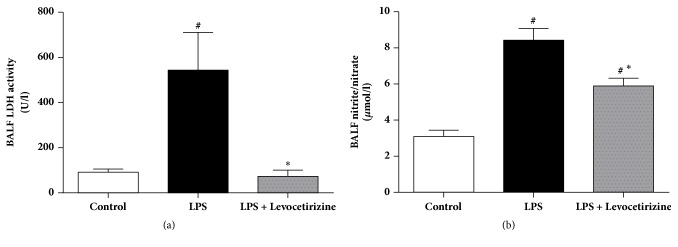
Effects of levocetirizine on lactate dehydrogenase (LDH) activity (a) and total nitrite/nitrate (b) in bronchoalveolar lavage fluid (BALF) of LPS-challenged rats. Results are shown as means ± SEM of 6 rats in each group. *∗* and # are significantly different (P < 0.05) versus LPS-treated and control groups, respectively.

The BALF of LPS-administered rats exhibited significant increases of LDH activity (P < 0.05, Figure 4(a)) and total NOx (P < 0.0001, Figure 4(b)) compared to control group. Levocetirizine restored normal BALF level of LDH (P > 0.05 relative to control group) and attenuated the increased concentrations of NOx in LPS group (P < 0.01 vs. both LPS and control groups).

### 3.5. Lung Oxidative Status (Figure 5)

In LPS group, lung tissues exhibited significantly higher MDA levels (P < 0.01 vs. control) and significantly lower GSH (P < 0.0001 vs. control) and SOD activity (P < 0.001 vs. control). Levocetirizine administration abolished LPS-induced changes in MDA and SOD levels. However, lung GSH levels in LPS group were not altered by levocetirizine treatment.

### 3.6. Lung and BALF TNF-*α* Levels

**Figure 6:**
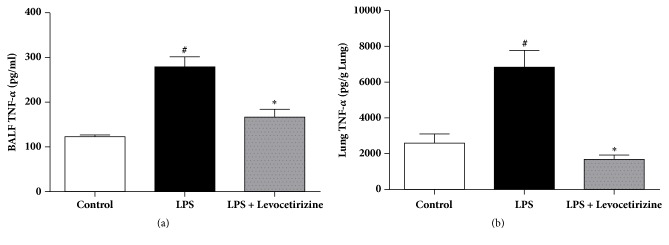
Effects of levocetirizine on tumor necrosis factor-*α* (TNF-*α*) levels in bronchoalveolar lavage fluid (BALF, panel (a)) and lung tissue (panel (b)) of LPS-challenged rats. Results are shown as means ± SEM of 6 rats in each group. *∗* and # are significantly different (P < 0.05) versus LPS-treated and control groups, respectively.

LPS-treated rats demonstrated significant increases of the levels of the proinflammatory TNF-*α* in the BALF (P < 0.0001) and lung homogenates (P < 0.001) relative to control group. LPS-mediated elevations of BALF and lung TNF-*α* level were significantly improved by prior treatment with levocetirizine (Figure 6).

### 3.7. Lung Histology

**Figure 7:**
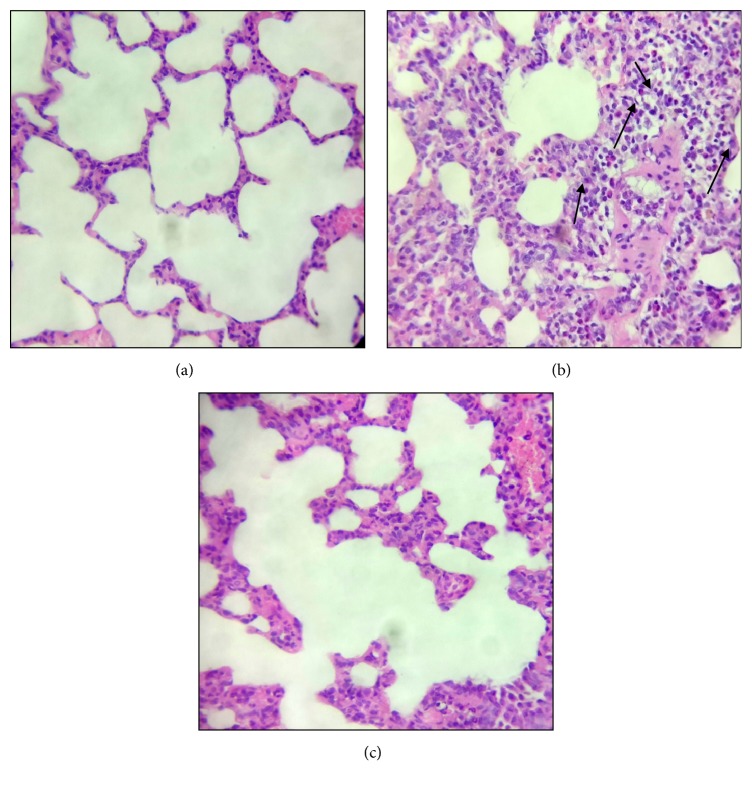
**Photomicrographs (H&E stain, 400 x) of lung tissues from control (a), LPS-challenged (b), and levocetirizine-pretreated (c) rats**. Arrows denote inflammatory cell infiltration.

**Figure 8:**
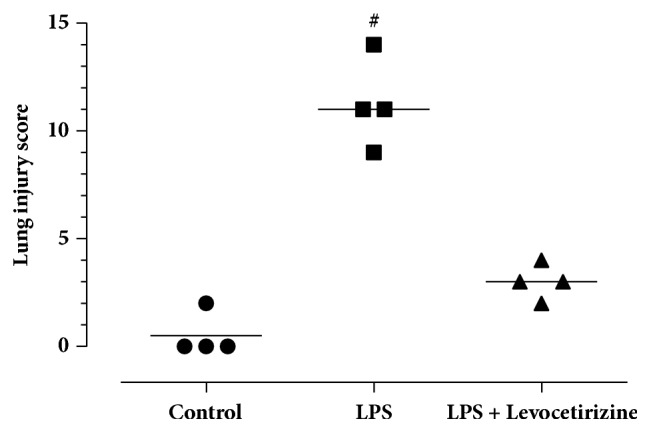
Total lung injury scores in the study groups. The median of each group is shown. # is significantly different (P < 0.05) versus the control group.

Control rats demonstrated normal pulmonary histology (Figure 7(a)). Examination of lung tissues of LPS-administered animals (Figure 7(b)) revealed substantial histopathological changes, including interstitial and alveolar edema, hemorrhage, alveolar wall thickening, and neutrophil infiltration into the interstitial and alveolar spaces when compared to the control group. These histological alterations were markedly ameliorated in the levocetirizine-pretreated rats (Figure 7(c)). A semiquantitative analysis of the histological changes in lung tissues in all groups is shown in Figure 8. The total lung injury scores in LPS-treated rats (9-14; median = 11) were significantly higher (P < 0.01) when compared with normal rats (0-2; median = 0). The lung injury scores in LPS + levocetirizine group (2-4; median = 3) were less than those in LPS-administered rats, indicating a protective effect of levocetirizine pretreatment against LPS-induced histopathological alterations.

## 4. Discussion

An intraperitoneal LPS challenge was used to provoke lung inflammation and injury in rats in the current study. Intratracheal [7], intravenous [19], and intraperitoneal [18] administrations of LPS to rats have been reported to elicit experimental ALI that closely resembles human ALI/ARDS. In the present study, LPS-challenged rats exhibited major features of ALI, including (i) a significant elevation of lung W/D ratio (indicating tissue edema), (ii) a marked increase in BALF total cells (indicating infiltration of activated inflammatory cells into lung tissue), (iii) an increased BALF level of total protein content (indicating enhanced alveolar-capillary membrane permeability of the barrier), and (iv) characteristic histopathological alterations in lung tissues. The findings of the current study suggest that levocetirizine pretreatment may mitigate ALI via suppression of oxidative damage and inflammation.

LPS administration elicited a marked oxidative stress in lung tissues, which showed an increase of lipid peroxidation and marked reductions in SOD activity and GSH levels. Oxidative injury is a key player in the development of ALI [7, 10]. Recruitment and activation of inflammatory cells during lung injury result in the overproduction of ROS, which interact with various cellular macromolecules and ultimately lead to disruption of lung function parameters [25, 26]. Natural host defenses fail to restore oxidant/antioxidant balance despite ALI-induced activation of antioxidant enzyme systems [27].

In the present study, levocetirizine offered antioxidant influences in LPS-challenged rats. Lung lipid peroxidation was reduced and SOD activity was substantially elevated in rats that were pretreated with levocetirizine compared with the untreated LPS group. Supporting these findings, levocetirizine ameliorated high fructose diet-induced hepatic oxidative stress in rats‏ [16]. Paradoxically, levocetirizine failed to restore normal lung GSH concentrations.

Possible mechanisms that may mediate the antioxidative effects of levocetirizine include H_1_ receptor blockade. Histamine was shown to stimulate release of hydrogen peroxide by primary bronchial epithelial cells via H_1_ receptor-dependent signaling [28]. Several H_1_-antihistamines diminished the production of ROS in neutrophils isolated from rat blood [29]. Moreover, it may be possible that levocetirizine inactivates ROS through direct scavenging activity or via activation of SOD, the only antioxidant enzyme that can scavenge superoxide [30]. Interestingly, it has also been shown that levocetirizine enhanced production of thioredoxin, a natural ROS scavenger, in hydrogen peroxide-stimulated macrophages [31]. Furthermore, levocetirizine may attenuate oxidant generation by reducing alveolar infiltration of inflammatory cells. Cetirizine inhibited recruitment and activation of inflammatory cells and suppressed production of reactive oxygen radicals, lipid mediators, and proinflammatory cytokines at sites of inflammation [32–35].

The beneficial influences of levocetirizine may be mediated via attenuation of LPS-induced increase of BALF NO level. The elevation of pulmonary NO was reported to contribute to ALI-associated inflammation, oxidative stress, and cytotoxicity [36, 37]. NO reacts with superoxide anion, generating peroxynitrite anion, which decomposes into highly reactive oxidative radicals [38]. Moreover, peroxynitrite modifies protein structure by reacting with various amino acids such as cysteine and tyrosine. These reactions impair cell signal transduction [39], resulting in apoptosis and dysfunction of microvascular endothelial barrier [40, 41]. Overproduction of lung NO in LPS-treated rats may be related to an enhanced expression of the inducible NO synthase (iNOS) [37, 42]. It remains to be investigated whether levocetirizine influences pulmonary iNOS expression in LPS-challenged rats. H_1_ receptor antagonists have been shown to inhibit NO production by LPS-stimulated murine macrophages via downregulation of iNOS protein expression [43]. Interestingly, the degree of inhibition of nitrite accumulation by H_1_ antihistamines correlated well with their degree of lipophilicity [43], which may explain failure of levocetirizine, a low lipophilicity H_1_ antihistamine [44, 45], to completely abolish LPS-mediated increase of pulmonary NO in the current investigation.

Lung inflammation occurs in response to injurious insults, which include bacterial endotoxemia and exposure to toxic chemicals [10, 18, 46]. The inflammatory response involves activation of several types of inflammatory cells, which release proinflammatory cytokines, such as TNF-*α* and interleukin (IL)-6. Secreted cytokines contribute to progression of inflammation via enhancement of expression of adhesion molecules on microvascular endothelium and stimulation of chemotaxis and activation of neutrophils, which release ROS, proteolytic enzymes, and additional cytokines [46–48].

In the current study, levocetirizine pretreatment attenuated LPS-induced inflammation. Rats preadministered with levocetirizine showed significant decreases in serum CRP, lung edema, BALF cell count, and BALF protein content, relative to the untreated LPS group. Additionally, histological assessment of lung tissues showed that levocetirizine reduced the diffuse neutrophil infiltration in lungs of LPS-treated animals, suggesting an anti-inflammatory influence of levocetirizine.

Levocetirizine attenuated LPS-induced increase of TNF-*α* level in BALF and lung tissue. This is consistent with other studies [8, 49]. TNF-*α* contributes to LPS-induced ALI via different mechanisms. TNF-*α* promotes the infiltration of neutrophils into lung [50]. Moreover, TNF-*α* increases ROS generation by neutrophils [51]. Therefore, the protective effect of levocetirizine against LPS-induced ALI is possibly mediated via suppression of the proinflammatory TNF-*α* in the lung tissue. The anti-inflammatory potential of levocetirizine may also be dependent on its ability to mitigate oxidative stress in the lung milieu since excessive production of ROS can trigger the expression of proinflammatory cytokines [7, 51].

LPS administration also resulted in cytotoxicity, as indicated by increased BALF levels of the cytosolic enzyme, LDH. Levocetirizine pretreatment attenuated the elevation of LDH level, implying a cytoprotective effect. Histopathological assessments of the lung tissues further emphasized the protective effects of levocetirizine against LPS-induced tissue damage.

The molecular mechanisms involved in the antioxidant and anti-inflammatory activities exerted by levocetirizine were not addressed in the present study. ‏Histamine concentrations have been shown to increase in BALF of rats with LPS-induced ALI [52], possibly via LPS induction of the histamine-forming enzyme histidine decarboxylase in the lung tissues [53, 54]. Histamine is reported to play contradictory roles in immune cell-driven inflammation depending on the histamine receptor subtype involved and consequently the distinct downstream regulatory pathways activated. It has been demonstrated that histamine increases the release of proinflammatory IL-6 by lung macrophages via H_1_ receptor activation [55], whereas it inhibits chemotaxis, phagocytosis, and production of TNF-*α* and superoxide anion via H_2_ receptors [56, 57]. Therefore, a possible explanation of the findings in the present study is that blockade of pulmonary H_1_ receptors by levocetirizine reduces LPS-induced lung inflammation, allowing histamine to exert unopposed anti-inflammatory roles via H_2_ receptors, which have been reported to mediate protective effects in LPS-induced tissue injury [58]. This speculation needs to be investigated in future research.

In the current study, levocetirizine was administered at a dose of 1 mg/kg/day, which corresponds to, based on dose conversion between rat and human [59], the recommended therapeutic dose in human, 10 mg/day. Moreover, administration of levocetirizine (1 mg/kg) in rats resulted in only 22.5% occupancy of the brain H_1_ receptors, indicating that levocetirizine, at this dose level, exerts effective peripheral H_1_ blockade without central adverse effects [21, 60]. Furthermore, levocetirizine (1 mg/kg) treatment exhibited anti-inflammatory and antioxidative effects in rats [16, 61, 62].

In conclusion, the present study suggests that levocetirizine, a nonsedating H_1_-receptor antagonist, ameliorated lung injury-associated vascular permeability, edema, and histopathological changes in LPS-challenged rats.

## Figures and Tables

**Figure 1:**
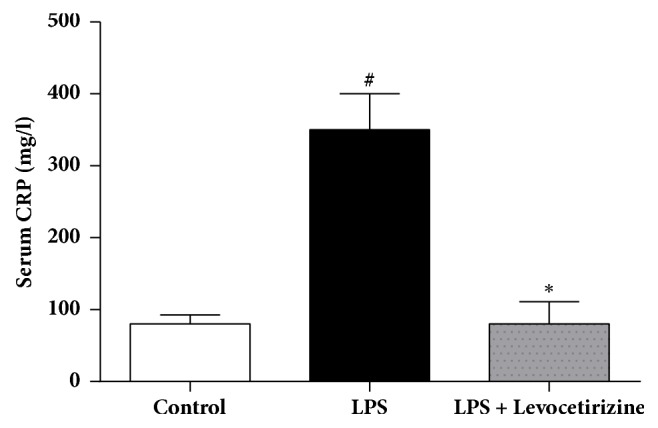
Effects of levocetirizine on serum C-reactive protein (CRP) levels in LPS-challenged rats. Results are shown as means ± SEM of 6 rats in each group. *∗* and # are significantly different (P < 0.05) versus LPS-treated and control groups, respectively.

**Figure 2:**
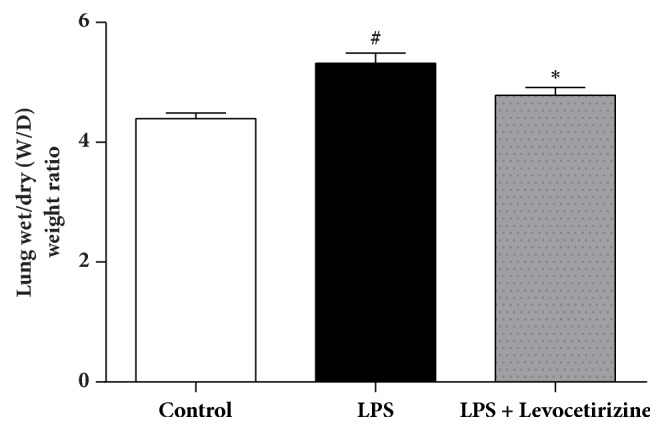
Effects of levocetirizine on lung wet/dry (W/D) weight ratio in LPS-challenged rats. Results are shown as means ± SEM of 6 rats in each group. *∗* and # are significantly different (P < 0.05) versus LPS-treated and control groups, respectively.

**Figure 5:**
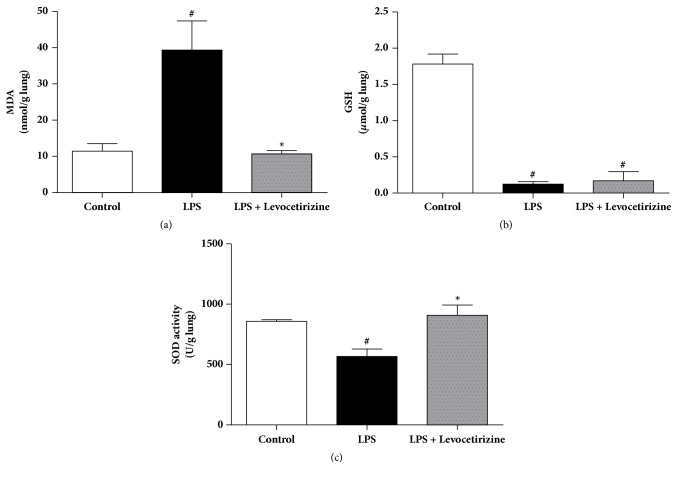
Effects of levocetirizine on lung oxidative stress in LPS-challenged rats. (a) malondialdehyde (MDA), (b) glutathione (GSH) and (c) superoxide dismutase (SOD). Results are shown as means ± SEM of 6 rats in each group. *∗* and # are significantly different (P < 0.05) versus LPS-treated and control groups, respectively.

## Data Availability

The data used to support the findings of this study are included within the article.
